# Durability and Self-healing Effects of Hydrogel Coatings with respect to Contact Condition

**DOI:** 10.1038/s41598-017-07106-x

**Published:** 2017-07-31

**Authors:** Chang-Lae Kim, Dae-Eun Kim

**Affiliations:** Center for Nano-Wear, School of Mechanical Engineering, Yonsei University, Seoul, 03722 Republic of Korea

## Abstract

The self-healing property of a hydrogel applied to a glass substrate as a thin polymer coating was assessed. The motivation was to develop a durable hydrogel coating that may be used to protect the surface of precision components from surface damage and scratches. The intrinsic swelling behavior of hydrogel fibers when they are exposed to moisture was exploited to attain the self-healing effect. The mechanical and self-healing properties of the dehydrated hydrogel coating by the freeze-drying process and the hydrated hydrogel coating that was reconstituted by the addition of water were analyzed. After conducting sliding tests with different loads and sliding distances, the wear area was hydrated with water to successfully induce self-healing of the hydrogel coating. It was also found that both the dehydrated hydrogel coating and the hydrated hydrogel coating had improved friction characteristics. In particular, the hydrated hydrogel coating had a much higher durability than the dehydrated coating.

## Introduction

Self-healing occurs when a material recovers by itself after mechanical damage. Such a property may be useful in cases where the material is easily damaged by sliding contact or physical deformation^1^. In this regard, various types of materials with durable self-healing properties have been developed. Additives used to heal fractures in a material were introduced by Sisomphon *et al*.^2^. Calcium sulfoaluminate-based additives with the ability to expand and crystalline additives were inserted into cracks in cement-based materials as a filling agent. Other concepts of self-healing using healing agents include the use of microcapsules and microvascular structures^3, 4^. Wang *et al*. reported that a self-healing agent embedded in a material enabled the material to recover by itself without the use of additives when the material was damaged^3^. However, microcapsules have a disadvantage in that they are designed for one-time use only. Moreover, in the case of microvascular structures, suggested by Hansen *et al*.^4^ as a way of overcoming the disadvantages of microcapsules, the manufacturing process required to design the special inner structure is relatively complex.

The self-healing methods described above commonly require a chemical healing agent that triggers a chemical reaction. However, there are many situations in which chemical reactions may be detrimental because of biocompatibility issues and environmental contamination. In order to overcome this problem, polymer-based materials are widely utilized for self-healing because they do not require an additional healing agent or have a complicated structure. The formation process is relatively simple and the damage can be healed without adding any chemical agent. A composite polymer based on urethane to heal fractures in a material using UV stimulus was introduced by Ling *et al*.^5^. However, the use of polyurethane is limited in biological applications because it releases toxic gases.

In this regard, collagens and hydrogels with self-healing abilities would be useful to minimize or avoid such problems^6^. These kinds of materials are generally utilized in biomedical applications such as tissue engineering and drug delivery systems because they are biocompatible and biodegradable^7^. Collagens and hydrogels also swell when hydrated with water^8, 9^, and the physical and mechanical properties of these materials as a function of this swelling have been investigated^10^. Especially, the correlation between the durability and swelling behavior of collagens and hydrogels with respect to the fiber density, pH, and additives have been studied^11, 12^.

Although collagens can be applied to various fields, they have limitations in that they are more sensitive to temperature, humidity, and pH than hydrogels because the main raw components of a collagen material come from the tissues of living creatures. Thus, hydrogels, which can be artificially chemically synthesized, would be more useful for a wide range of applications. In particular, the self-healing ability of hydrogels may be utilized to develop functional biocompatible coatings in order to protect the surfaces of precision components. It is thus necessary to assess the durability of hydrogels as thin coatings. Many studies on the bulk hydrogels for application in the field of biotechnology such as cell culture, drug delivery, and implants have been conducted^13–16^. However, there are only a few reports on the mechanical and tribological characteristics of hydrogel coatings in the form of thin films.

In this study, a self-healing hydrogel coating was formed on a glass substrate to protect the surface from damage due to contact stress. Experiments were performed to assess the physical, chemical, and mechanical properties of the hydrogel coatings. To understand the wear mechanism, sliding contact wear tests on the hydrogel coating were conducted and the friction characteristics were analyzed through our previous experimental methods^17–21^. The amount of wear on the hydrogel coating surface with respect to the contact conditions was compared, and the self-healing abilities of the hydrogel coating hydrated with water were verified. The durability of the hydrogel coating to maintain its self-healing effect was assessed through long-term sliding tests.

## Results

### Physical, chemical and mechanical properties of hydrogel coating

**Figure 1:**
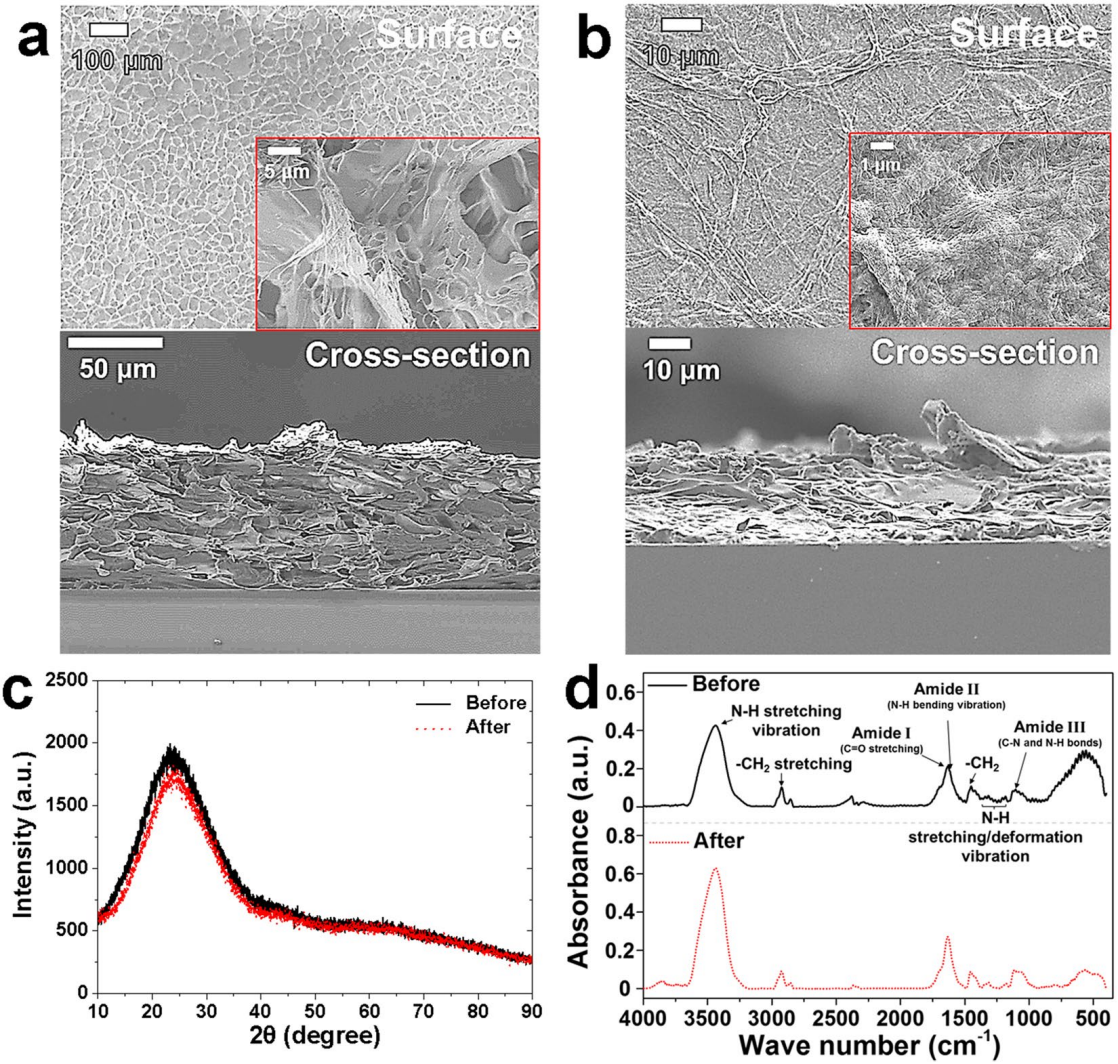
Structure and properties of hydrogel coatings SEM images of the surface morphology of the hydrogel coatings after freeze drying (**a**) before and (**b**) after hydration. (**c**) XRD data and (**d**) FTIR spectra for hydrogel coating before (black solid line) and after (red dash line) hydration.

The surface morphology of the dehydrated hydrogel coating before and after hydration was investigated using the scanning electron microscope (SEM, JEOL-7800F, JEOL Inc.) measurement, and the respective SEM images are shown in Fig. 1a and b. The hydrogel coating had irregularly entangled fibers with diameters from several tens to hundreds of nanometers and a porous structure^22^. The thickness and roughness of the dehydrated hydrogel coating were approximately 70 µm and 2.1 µm, respectively (Fig. 1a, Table 1). After wetting the dehydrated fibers with water, the surface morphology of the hydrated hydrogel coating was compared to that of the dehydrated hydrogel coating. It was found that the hydrated hydrogel coating formed a different structure upon naturally drying in an atmospheric environment. The shape and size of the hydrogel fibers changed after hydration, and therefore the surface morphology of the hydrogel coating after hydration was quite different (Fig. 1b). It was also confirmed that the thickness and roughness of the hydrated hydrogel coating were approximately 25 µm and 0.3 µm, respectively.

**Table 1:** Hardness, elastic modulus, stiffness, thickness and surface roughness of the hydrogel coating specimen before and after hydration.

Hydrogel coating	Hardness (MPa)	Elastic modulus (MPa)	Stiffness (mN/µm)	Thickness (µm)	Roughness (µm)
Before hydration	0.1~0.3	6.2~7.8	0.5~1.8	70	2.1
After hydration	0.3~0.9	10~23	2.0~3.6	25	0.3

The structure of the hydrogel coating before and after hydration was analyzed by an X-ray diffraction (XRD, Rigaku SmartLab) measurement. The hydrogel coating before and after hydration showed non-crystalline diffraction patterns at 2θ = 24°, with no high-intensity peak. This indicated that the crystallinity of the hydrogel coating did not change with respect to hydration. However, as shown in Fig. 1d, a distinct variation in the chemical condition of the hydrogel coating with respect to hydration was determined using the FTIR spectrometer (Vertex 70, Bruker Inc.). At similar positions on both coatings, typical bands for amides I, II, and III were observed^23^. In the case of the dehydrated hydrogel coating, amide I was confirmed to be the C=O stretching vibration of the carbonyl group at 1655–1638 cm^−1^. At 1618–1611 cm^−1^, the deformation vibration of the –NH_2_ groups was measured as amide II. For amide III, C-N and N-H bonds were detected at 1116 cm^−1^. Moreover, the deformation vibration of the –NH groups was measured at 1421 cm^−1^ and 1177 cm^−1^. The stretching vibration of the –NH groups was observed at 1320 cm^−1^. In addition, it was considered that the absorption peak at 3436 cm^−1^ showed an N–H stretching vibration related to the polymerization of the hydrogel, and the peaks at 2923 cm^−1^ and 1458 cm^−1^ appeared to be attributable to −CH_2_ on the polymeric chains. The absorption peak of amide I (C=O stretching vibration) in the hydrated hydrogel coating shifted to lower wavenumbers of 1637–1629 cm^−1^. This frequency shift resulted from the interaction between the C=O group and the water molecules^24, 25^. At 3400 cm^−1^, the N–H stretching vibration could be distinguished. The peak of the N–H stretching vibration became broader and the intensity increased due to hydrogen bonding after hydration. Based on these results, it was supposed that the molecular orientation of the hydrogels was altered^26^.

The mechanical properties of the hydrogel coating were assessed using indentation tests. As shown in Table 1, it was confirmed that hydration had a significant effect on the mechanical properties of the hydrogel coating. The hardness, elastic modulus, and stiffness of the hydrated hydrogel coating are 2.5~3 times higher than those of dehydrated hydrogel coating.

### Friction and wear characteristics of hydrogel coating

**Figure 2:**
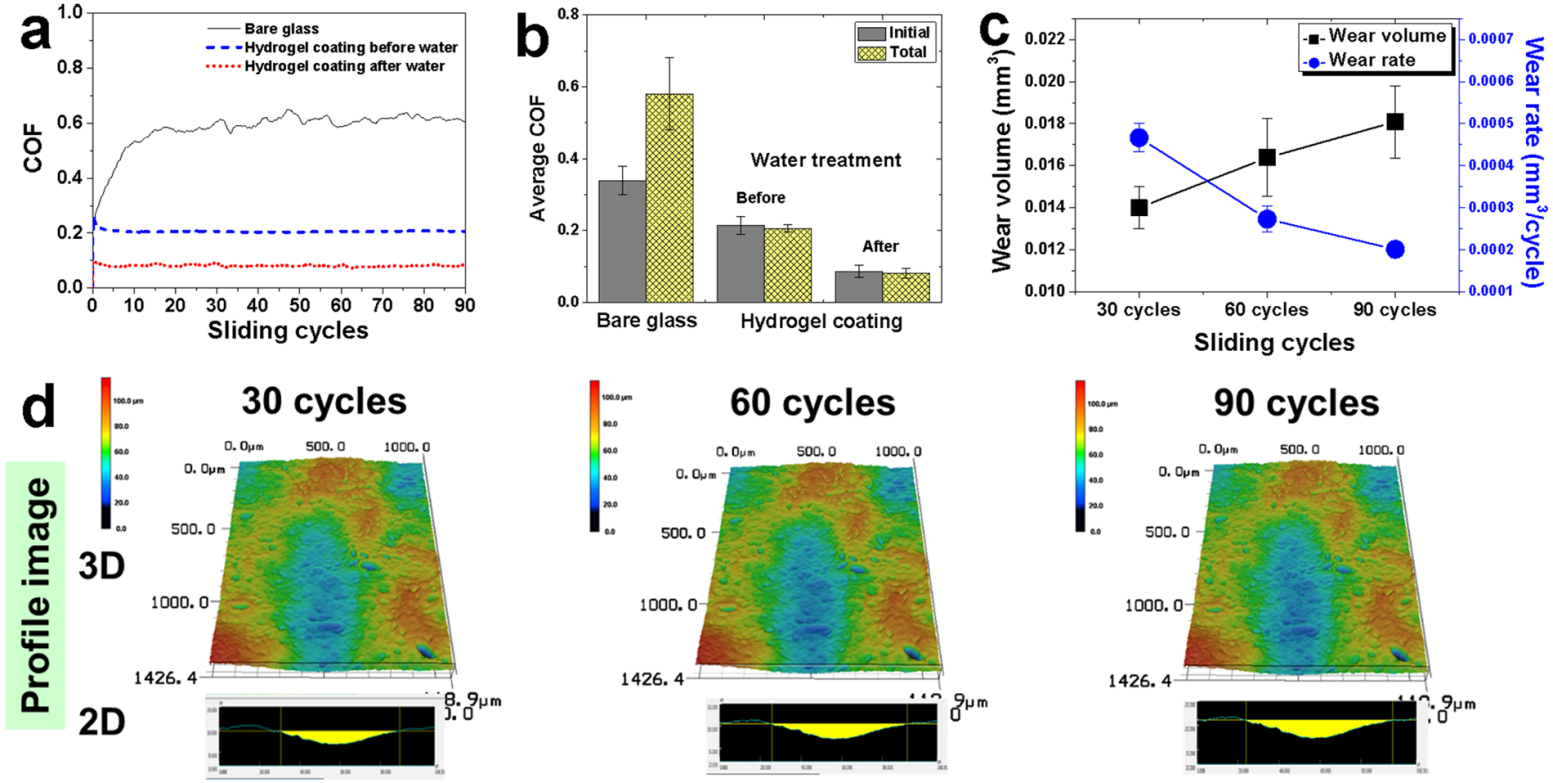
Friction and wear characteristics of hydrogel coatings. Average COF of glass and hydrogel coating specimen before and after hydration with respect to (**a**) the number of sliding cycles and (**b**) the initial and total stages of the sliding test. (**c**) Average wear volume and wear rate of the hydrogel coating before hydration after 90 cycles of sliding. (**d**) Confocal microscope 2D/3D profile images of wear track of the dehydrated hydrogel coating after every 30 cycles.

The friction and wear characteristics of the hydrogel coating were assessed by reciprocating sliding tests. The average coefficient of friction (COF) of the hydrogel coating under an applied load of 20 mN as a function of the number of sliding cycles is shown in Fig. 2a. Also provided in Fig. 2b is the average COF of the glass substrate under the same conditions. The average COF of the glass initially increased steadily. The average COF varied from 0.25 to 0.6, with a relatively large standard deviation, whereas the hydrogel coating showed a much lower COF and standard deviation than the glass. However, the average COF of the hydrogel coating was initially relatively high, 0.25. It then decreased to approximately 0.2 after several cycles and remained steady.

**Figure 3:**
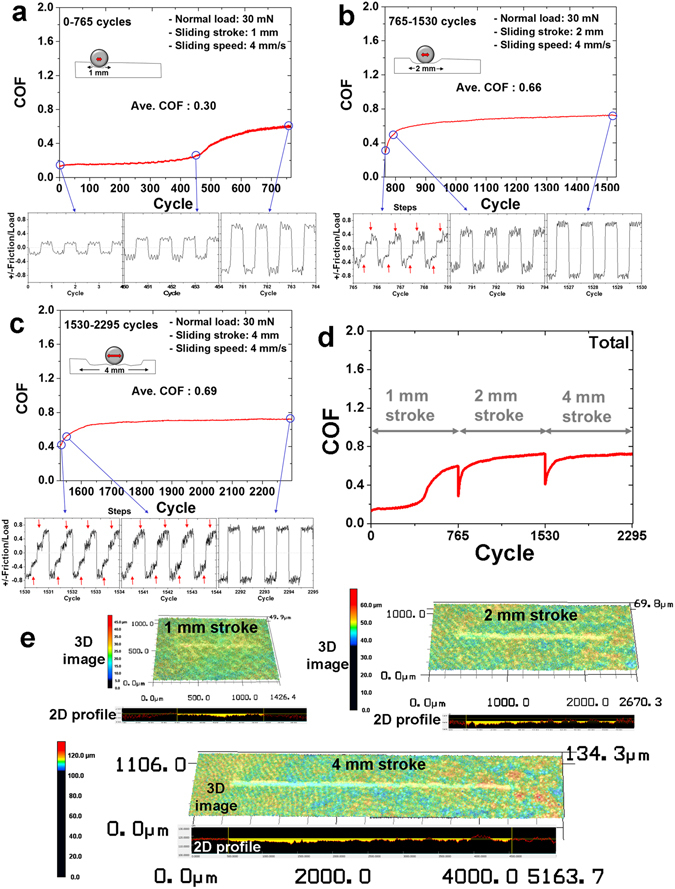
Variation of friction and wear characteristics of dehydrated hydrogel coating with respect to the sliding stroke length. Average COF and (±) friction/load of hydrogel coating as a function of the number of sliding cycles for stroke lengths of (**a**) 1 mm, (**b**) 2 mm, and (**c**) 4 mm. (**d**) Total average COF of the hydrogel coating over 2295 cycles. (**e**) Confocal microscope 2D/3D profile images of wear track with respect to the sliding stroke length. Red arrows indicate the different steps of the friction force signal during the half-cycle.

The reason for the initial variation in COF and the subsequent steady state behavior was investigated. In order to further analyze the variation of the frictional force, the sliding stroke was increased with a step, 1 mm, 2 mm and 4 mm of length, during the test conducted with an applied load of 30 mN. The COF with a 1-mm-long sliding stroke significantly increased from 0.18 to 0.6 (Fig. 3a). The average COF of the hydrogel coating over all cycles was 0.3. The COF can be described as the variation of the frictional force divided by the normal load with respect to the sliding direction, indicated using plus (+) and minus (−) signs for each half-cycle. The amplitude of the force variation rapidly increased when the number of cycles was between 400 and 600. Although the fluctuation of the force signal increased slightly with respect to the cycle number, it remained entirely steady for the 1-mm sliding stroke length over 765 cycles. After finishing the sliding test with the 1-mm-long stroke, testing was continued with a 2-mm-long sliding stroke under the same conditions of load, sliding speed, and number of cycles. The COF was initially low, being approximately 0.3. It then increased significantly to 0.6 over approximately 100 cycles (Fig. 3b), reaching 0.72. The average COF over all the cycles was 0.66. It was confirmed that a gap in the force signal was generated every half-cycle, and thus the signal appeared to be stair-like over the first several cycles.

In detail, the force signal of the half part during the half-cycle was relatively high, with a large deviation. However, the force signal and deviation of the other half part of the half-cycle were smaller than those of the first half part. The force signal during the half-cycle resulted from changes in the sliding stroke length. The half part of the half-cycle was deformed during the sliding test with the 1-mm-long sliding stroke length and the other half part of the half-cycle was the undamaged surface of the hydrogel coating. Therefore, in the sliding test with the 2-mm-long sliding stroke, the tip slid on different surfaces, thus generating the frictional signals described above. In other words, the friction force was relatively high when the tip came into contact with the compressed surface of the wear track, which was half of the 2-mm sliding stroke because the contact area between the tip and the compressed surface was large. On the other hand, when the tip passed across the undamaged surface, which was the other half of the 2-mm-long sliding stroke, the contact area between the tip and the intact surface was small, and so the frictional force decreased to a value similar to that of the initial cycles of the first sliding test with the 1-mm-long sliding stroke. As the number of sliding cycles increased, the stair-like gap in the friction signal during the half-cycle disappeared as the undamaged part of the 2-mm-long sliding stroke became compressed, which resulted in an increase in the contact area, similar to what occurred with the damaged part of the 1-mm-long stroke. As the testing progressed, the high fluctuation of the force signal decreased with respect to the increase in the number of sliding cycles.

After the sliding test with the 2-mm-long stroke for 765 cycles, the sliding stroke length was increased to 4 mm and the testing was continued under the same conditions of load, sliding speed, and number of cycles. The COF of the hydrogel coating varied from 0.45 to 0.65 over approximately 100 cycles (Fig. 3c). The value of the COF then increased slightly to 0.72. The average COF of the test with the 4-mm-long sliding stroke over all cycles was 0.69, which was quite similar to the results of the test with the 2-mm-long sliding stroke. There was also a gap in the force signal during the half-cycle at the initial stage. The stair-like gap of the force signal during the half-cycle of the test with the 4-mm-long sliding stroke was larger than that of the test with the 2-mm-long sliding stroke. The force signal of the half part during the half-cycle was high, similar to the final part of the force signal for the test with the 2-mm-long sliding stroke. However, the force signal of the other half part was low, which was similar to the force signal when the tip came into contact with the undamaged surface of the hydrogel coating. The contact area between the tip and the compressed surface in the wear track was much larger than that when the tip came into contact with the undamaged surface of the hydrogel coating for the first time. The variation of contact area resulted in the increase of the signal gap during the half-cycle. After 10 sliding cycles, the stair-like gap of the force signal during the half-cycle decreased because the undamaged surface of the hydrogel coating, which originally showed a low force signal, became compressed. Finally, after several hundred sliding cycles, the stair-like gap of the force signal during the half-cycle disappeared, and the fluctuation of the force signal decreased. Figure 3d shows the entire COF variation of the hydrogel coating with respect to the sliding stroke length.

The wear characteristics of the hydrogel coating were analyzed by visualizing the generation of the wear track during the sliding test conducted under a normal load of 20 mN. The wear track was observed every 30 cycles using a 3D laser scanning confocal microscope (VK-X200, Keyence Co.) which as installed above the tribotester. A two-dimensional (2D) profile and three-dimensional (3D) scanning images of the wear track were obtained (Fig. 2d). From these data, it was confirmed that the cross-sectional area of the wear track increased slightly with respect to the number of sliding cycles. The wear volume was obtained by multiplying the cross-sectional area of the wear track by the sliding stroke length. Although the wear volume of the hydrogel coating steadily increased every 30 cycles, the wear rate, which was calculated by dividing the wear volume by the number of sliding cycles, decreased (Fig. 2c). It was supposed that there was severe deformation of the hydrogel coating during the initial cycles because there were many pores among the entangled fibers. The hydrogel fibers were almost completely compressed after 30 cycles.

After the sliding test performed under a load of 30 mN with respect to the sliding stroke, the wear track was observed by confocal microscopy (Fig. 3). Each sliding test with the different sliding stroke lengths was conducted for 765 cycles. This means that the first wear section, which was generated in the first test with the 1-mm-long sliding stroke, was subjected to a total of 2295 sliding cycles. In the case of the 2-mm-long sliding stroke, the second wear section, which was newly formed because of the increased sliding stroke length, was subjected to repetitive sliding for 1530 cycles. Finally, the third wear section, which was newly generated after the sliding test with the 4-mm-long sliding stroke, experienced repeated sliding for 765 cycles. The surface and 2D profile images of the wear track on the hydrogel coating after the sliding tests were observed by confocal microscopy (Fig. 3e). It was confirmed that different wear depths and widths were generated in the same wear track. The average wear depth and area of the first wear section were 2.5 µm and 105 µm^2^, respectively (Fig. 3e). The average wear depth and area of the second wear section were 21.6 µm and 63 µm^2^, respectively, whereas those of the third wear section were 1.1 µm and 30 µm^2^, respectively. The wear characteristics of the ball used as the counter tip were also analyzed. It was found that there was no evidence of damage to the ball surface, which was attributed to the significantly higher hardness of the ball compared with that of the hydrogel coatings.

### Self-healing effect of hydrogel coating

**Figure 4:**
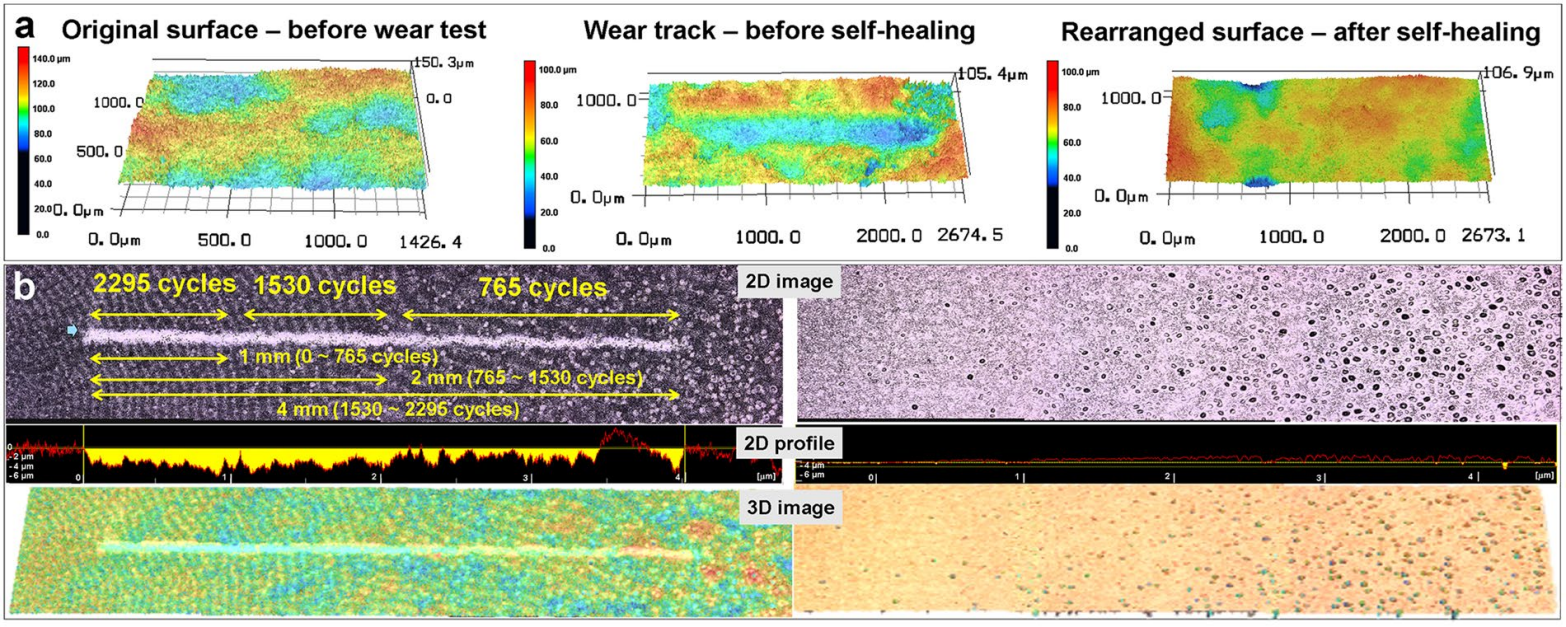
Confocal microscope 2D/3D images of hydrogel coating before/after self-healing. (**a**) Original surface, showing the formation of the wear track after the sliding test under an applied load of 20 mN over 90 cycles and the reconstituted surface of the hydrogel coating after self-healing. (**b**) Total wear track formed on the surface after the sliding test under an applied load of 30 mN over 2295 cycles with respect to the different sliding stroke lengths (1 mm, 2 mm, and 4 mm) and the reconstituted surface of the hydrogel coating after self-healing.

The self-healing property of the hydrogel coating was investigated after the friction and wear tests. For this purpose, the wear track generated on the hydrogel coating after the sliding test was compared to the surface after self-healing (Fig. 4a and b). The main principle of the self-healing characteristics of the hydrogel is its ability to swell when hydrated with water. To heal the wear track generated on the hydrogel coating surface, water was used as a mediator. The compressed area of the wear track disappeared with swelling of the hydrogel fibers when a small quantity of DI water was dropped onto the wear track.

It was found that there was significant variation of the surface morphology during the self-healing process (Fig. 4a and b). The self-healing effect was visible immediately after hydration with water, with simultaneous alteration of the surface morphology. Such changes in the morphology of the hydrogel coating affected the mechanical properties. To assess the durability of the coating, additional sliding tests were conducted with a load of 20 mN for 90 cycles and the contact sliding area was observed every 30 cycles by confocal microscopy. It was interesting to note that there was no sign of wear. Moreover, the COF of the self-healed hydrogel coating remained relatively constant at 0.08 over 90 cycles. The sliding test was then carried out under the same load for 300 cycles. The average value and variation of the COF were quite similar to those of the first sliding test conducted for 90 cycles. Every 100 cycles, the variation of the surface morphology was observed by confocal microscopy. However, no evidence of wear could be found on the surface of the hydrogel coating hydrated with water. Thus, to assess the durability of the hydrogel coating, additional tests under a higher normal load of 30 mN were performed for 300 cycles. After the test, the surface morphology of the hydrogel coating was observed by confocal microscopy. Again, there was no wear found on the surface of the hydrogel coating. Thus, it was confirmed that hydration triggered self-healing of the damaged part of the hydrogel coating. Furthermore, variation of the surface morphology by water also significantly affected the mechanical properties of the coating, greatly increasing its durability.

## Discussion

Based on the experimental results, it was suggested that hydrogel coatings possess self-healing properties that may be utilized to protect the surfaces of precision components that need to be biocompatible. The self-healing ability of the hydrogel coating was verified by the hydration process, which improved the mechanical and physical properties and enhanced the durability of the hydrogel coating. The variation of the surface morphology of the hydrogel coating after water hydration was observed by SEM analysis (Fig. 1). The significant variation of the surface morphology of the hydrogel coating was because of the sensitivity of the hydrogel fibers to water^27^.

From the results of the XRD analysis, it was confirmed that the hydrogel coating had an amorphous structure, which is usually found in polymeric materials. The orientation of the hydrogel coating with respect to hydration was assessed by FTIR analysis (Fig. 1d). There was a slight difference in the orientation of the hydrogel coating before and after hydration. The chemical structure of the amide, which was the main element of the hydrogel coating, was in the form of carbon double-bonded to oxygen (C=O) and single-bonded to nitrogen (C-N). There are two major properties of the amide compound. The first is its resonance structure, which results in a reduction of the double bond between C and O. Therefore, the absorption peak of the C=O stretching vibration in the amide compound was detected at less than 1700 cm^−1^, although it is usually observed above 1700 cm^−1^. Hydrogen bonding is the second major property of amide compounds. Because of this hydrogen bonding, the peak of the N–H stretching vibration broadened, and the absorption intensity slightly increased to 3400 cm^−1^. Moreover, there are different types of amides, i.e., primary, secondary, and tertiary amides, thus changing the number of H bound to the N of the amide, i.e., 2, 1, and 0, respectively. The primary amide has symmetric and asymmetric N–H stretching bands at approximately 3180 cm^−1^ and 3350 cm^−1^, respectively. The secondary amide has only the one N–H stretching band at approximately 3400 cm^–1^. In this study, the hydrogel coating had the secondary amide, and thus the absorption peak of the N–H stretching vibration in the hydrogel coating was detected at 3436 cm^−1^. In addition, the wavenumber of the N–H groups decreased because of hydrogen bonding after hydration. In other words, the frequency shift of N–H stretching increased the hydrogen bonding. In general, the absorption peak of the C=O group was close to 1640 cm^−1^. The wavenumber range of this peak was measured to be 1655–1638 cm^−1^. After hydration, the peaks of C=O stretching of the hydrogels shifted to higher wavenumbers. This shift of the wavenumber also resulted from the hydrogen bonding between the water molecules and the hydrophilic parts of the polyacrylamide chains^28^. Moreover, the typical peak of the acrylamide monomer at ~890 cm^−1^ was not detected^28, 29^. From this result, it was confirmed that the hydrogel was polymerized.

After self-healing by means of hydration, the reconstituted hydrogel coating had improved mechanical properties. This was expected to have resulted from the variation in the physical and chemical properties of the hydrogel coating after hydration. Above all, the surface roughness and the thickness of the hydrogel coating decreased considerably, by approximately three times and seven times, respectively. Therefore, the hydrogel coating reconstituted with a relatively smooth and dense structure as a result of the swelling effect of the hydrogel fibers^6, 8–10, 27^. In terms of the thickness, the capillary action of water in the hydrogel fibers caused compression in the fibrous structure^30^. Essentially, it was postulated that the mechanical properties of the hydrogel coating were enhanced after hydration because of compression of the entangled hydrogel fibers.

The initial COF of the hydrogel coating decreased slightly after several cycles and then remained constant. The decrease in the COF resulted from the decrease in surface roughness as the hydrogel coating became compressed against the ZrO_2_ ball tip (Fig. 2a). It also decreased after hydration as compared to the value before hydration (Fig. 2a and b). The lower surface roughness and improved mechanical properties of the hydrated hydrogel coating as compared to the untreated coating was considered to be the main reason for the improved frictional characteristics of the hydrogel coating.

The amount of wear of the hydrogel coating was investigated using the width and depth of the wear track generated on the specimen after sliding tests under a normal load of 20 mN for 90 cycles. The wear tendency between the glass substrate and the hydrogel coating specimen was quite different. While the wear track generated on the glass surface was much deeper than the width, the width of the wear track generated on the hydrogel coating surface was much larger than the depth because the lower stiffness of the hydrogel coating. Thus, the force in the vertical direction was distributed over a relatively large contact area. The concept of controlling the surface stiffness in order to distribute the contact stress over a large area was introduced in previous studies using carbon nanotubes and collagen fibers^6, 31^.

**Figure 5:**
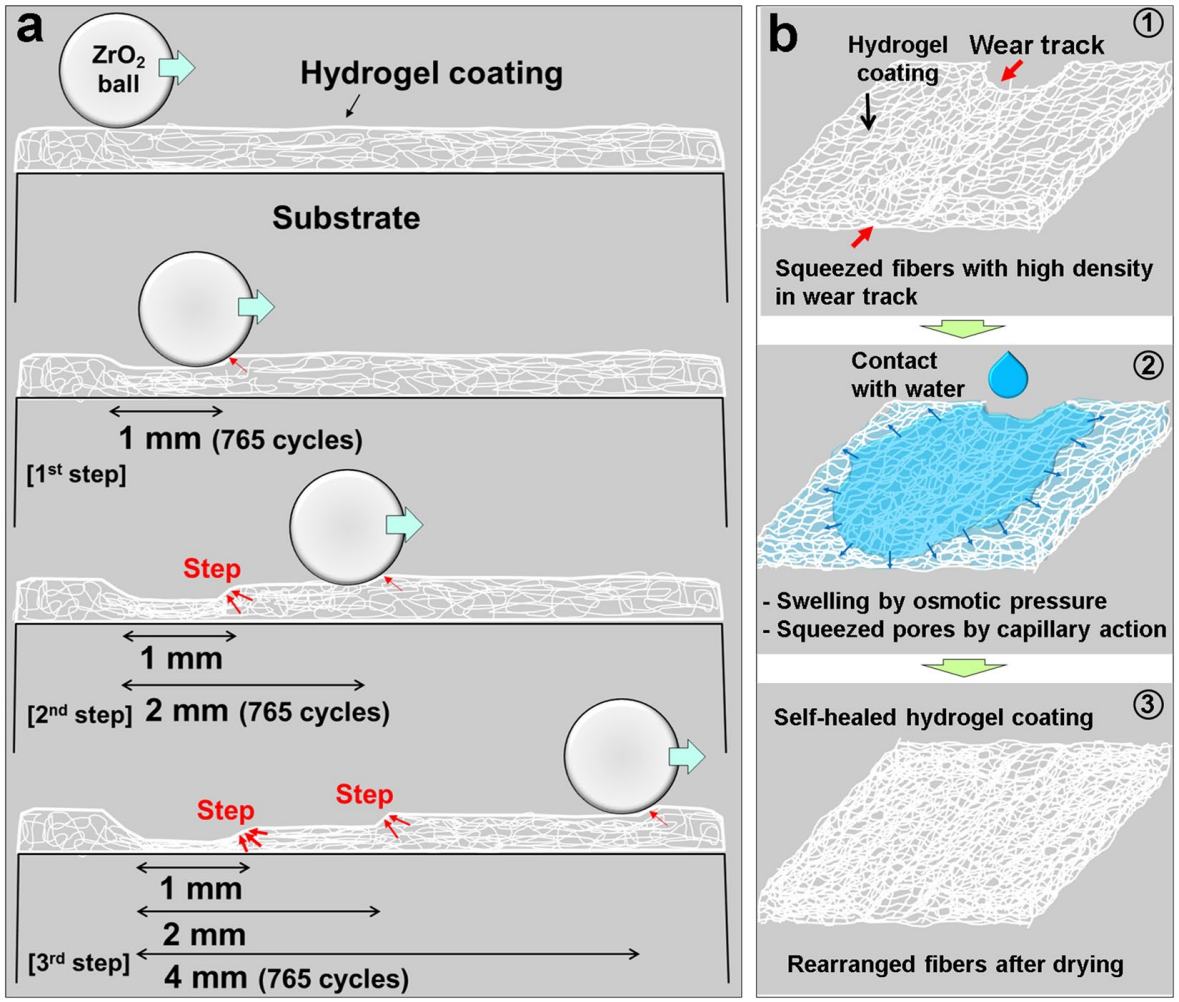
Mechanism and process of wear and self-healing of hydrogel. (**a**) Schematic of the wear track generation of the hydrogel coating with respect to the sliding stroke. Red arrows indicate the force concentration. (**b**) Schematic of the self-healing mechanism and process of wear track formation on the hydrogel coating surface. ① Wear track with compressed fibers generated on the hydrogel coating surface; ② After hydration, the fibers swell by osmosis and pores among the fibers are compressed by capillary action; ③ After drying, the fibers were reconstituted with self-healing. Red arrows indicate the wear track.

The wear tracks generated by continuous sliding tests with increasing sliding stroke length were analyzed by 2D profile data (Fig. 3e). The stroke ranges of 0–1 mm, 1–2 mm, and 2–4 mm were generated after the sliding tests under a 30 mN load for 2295, 1530, and 765 cycles, respectively. There were thus three steps with different wear depths and widths in a wear track with respect to the sliding stroke lengths (Fig. 5a). Naturally, the difference of the wear depth and width with respect to the sliding stroke length was generated due to the different numbers of sliding cycles. From this test, the variation of the frictional characteristics of the hydrogel coating with respect to the contact surface could be clearly understood. It was also confirmed that compression of the hydrogel fibers occurred during the initial sliding cycles. Although the different steps of the frictional force signal during the half-cycle disappeared after several tens of cycles, the step of each wear track remained throughout the cycles which allowed for detailed analysis of the wear behavior.

The self-healing effect of the wear track generated on the hydrogel coating was verified by observing the surface before and after hydration. The wear track on the hydrogel coating disappeared entirely after hydration. Even though there were different amounts of wear depth and width in the wear track, it recovered fully upon treatment with water. After self-healing, the physical and mechanical properties of the hydrogel coating were improved because of the variation of the coating structure. Essentially the variation in the coating morphology enhanced the friction and wear characteristics of the hydrogel coating. After identifying the physical, mechanical, and tribological behaviors of the hydrogel coating before and after self-healing, further analysis on the fundamental mechanism of the self-healing phenomenon was conducted. The main mechanism for the self-healing property was the swelling of the hydrogel fibers when they were hydrated with water (Fig. 5b).

The hydrogel membrane had a matrix-like structure and the thin membrane formed by hydrogel fibers has an irregular cell construction. The pores of the hydrogel structure acted as capillary channels, which transferred water molecules into the porous structure when the completely dried hydrogel was hydrated with water^30^. Then the microporous structure in the hydrogel fibers expanded by means of the water molecules, increasing the size of the hydrogel fibers. This phenomenon can be explained by the swelling mechanism, which has been reported in previous studies^6, 8–10, 27, 32–34^. After freeze drying, the hydrogel membrane was completely dried with relatively large pores (Fig. 1a). Furthermore, the sizes of the hydrogel fibers and membranes increased considerably after the hydration process (Fig. 1b). The capillary force between water molecules and fibers resulted in a decrease in the size and number of pores.

The swelling behavior of the hydrogel fibers is closely related to the self-healing mechanism of the wear track via hydration. During the sliding test, the wear track was generated on the surface of the hydrogel coating by the sliding action of the ball tip without severe material removal. Thus the surface damage was primarily in the form of compressed fibers, in which the contact pressure caused deformation of the porous structure of the hydrogel coating. The variation in the structure of the hydrogel fibers served to distribute the contact stress and frictional stress generated by the loading and sliding motion on the hydrogel coating surface. Figure 5b shows the main mechanism and process of self-healing of the hydrogel coating. The swelling of each fiber, which occurred by osmosis, filled the compressed fibers^35^. In other words, when the hydrogel fibers come into contact with water molecules, they expand through osmosis as per the Gibbs–Donnan effect, and the pores between the fibers shrink through the capillary process, and thus the hydrogel coating structure becomes more compact^30, 35, 36^. The hydrogel fibers cease to swell only when the osmotic force is equal to the morphological reaction force due to the elastic variation of the fiber membrane^6^. However, the self-healing effect would not be effective if the damage incurred to the hydrogel coating is severe. This was verified by a separate test in which the coating was scratched manually using a sharp metallic tip until the coating was totally penetrated. It was found that the swelling effect of the hydrogel coating after hydration was not sufficient to recover the original surface state as expected. Thus, the self-healing effect becomes ineffective when the hydrogel fibers are severely damaged through rupture and tearing.

Water added to the compressed fibers of the wear track can enter adjacent undamaged fibers through capillary action. During this process, empty pores among the uncompressed fibers shrink while the fibers expanded. In other words, the water molecules triggered swelling of the hydrogel fibers, thinning the coating because of the capillary force between the water molecules and the fibers. The wear track generated on the hydrogel coating after repeated contact sliding motion recovered after hydration. During self-healing, the mechanical properties of the coating were enhanced because of changes to the hydrogel coating structure by water molecules. Thus, even after the sliding test under a higher normal load of 30 mN for 300 cycles, there was no generation of a wear track on the hydrogel coating after hydration. The wear characteristics of the hydrogel coating after hydration were better than those of the coating before hydration.

## Conclusions

The physical, chemical, and mechanical properties of a hydrogel coating that was fabricated on a glass substrate were investigated. After hydration, the mechanical properties of the hydrogel coating were considerably improved. The hydrogel coating after the freeze drying process showed outstanding self-healing of the wear track formed on the coating surface. The self-healing process by means of swelling and capillary behavior had a significant effect on the physical and mechanical properties of the hydrogel coating. It was found that the resistance to surface damage of the hydrogel coating could be significantly enhanced by hydration. It is expected that hydrogel coatings may be utilized as a biocompatible protective coating against surface damage and wear for precision components.

## Methods

### Fabrication of hydrogel coating

The hydrogel coating was formed on glass using a polyacrylamide (pAAm) solution. First, ammonium persulfate (AP) powder [(NH_4_)_2_S_2_O_8_, assay ≥98.0%, Sigma-Aldrich, Inc.] was dissolved in deionized (DI) water in a 1:9 weight ratio. The AP solution acted as the initiator. Then, an N,N′-methylenebisacrylamide solution (C_7_H_10_N_2_O_2_, 2% in ultra-pure water, Sigma-Aldrich, Inc.), used as the cross-linker, and an acrylamide solution (C_3_H_5_NO, 40% in ultra-pure water, Sigma-Aldrich, Inc.), used as the monomer, were mixed in a 1:1 volume ratio with DI water to fabricate a bis-acrylamide solution. The pAAm solution, i.e., the hydrogel solution, was blended with the AP solution and the bis-acrylamide solution. This led to the formation of a gel by means of a catalyst, which was an N,N,N′,N′-tetramethylethylenediamine (TEMED) solution (C_6_H_16_N_2_, assay ~99%, Sigma-Aldrich, Inc.). The TEMED solution, which was a tertiary amine base, helped generate free radicals from the ammonium persulfate, which in turn helped polymerize the acrylamide and bis-acrylamide, thus forming a gel matrix. For this study, 5 µL of the AP solution was mixed with 1,125 µL of the bis-acrylamide solution that was compounded with 375 µL of the cross-linker solution, the monomer solution and DI water, respectively. The resulting hydrogel solution was transformed to a gel-type state using 1 µL of the TEMED solution. A small amount of the hydrogel was then dropped onto the glass substrate and pressed by a glass plate to form a thin hydrogel coating. The top glass plate was cautiously detached after several minutes for solidification of the film. The hydrogel coating formed on the glass substrate was almost transparent.

The hydrogel coating was dehydrated by freeze drying in a freeze-drying machine (FDU-2110, Tokyo Rikakikai Co., Ltd.). The dehydration process was conducted for one day at a pressure of 7 Pa and a temperature of −80 °C. Sublimation was the main mechanism of freeze drying, and it led to the formation of a porous structure with many voids in hydrogel coatings^37–39^. Thus, a fibrous structural coating was formed after freeze drying the hydrogel coating. The dehydrated hydrogel coating was treated by water. After this hydration process, the hydrated hydrogel coating was dried at an atmospheric environment.

### Analysis of hydrogel coating properties

The SEM measurement was utilized for analysis of the surface of hydrogel coatings. Moreover, the cross section of hydrogel coating was observed by the SEM in order to measure the thickness of the coating. For measurement of the surface roughness of hydrogel coating, a three-dimensional (3D) profiler (Dektak XT, Bruker Inc.) was used. The crystal structure of the hydrogel coatings was analyzed by XRD measurement. The measurement of the conformational change in hydrogel coating before and after self-healing was conducted by the FTIR spectrometer.

The indentation tests on the hydrogel coating were performed in order to measure its mechanical property as shown in Table 2. The indenter tip that was made of a stainless steel (SUS 304) ball with a diameter of 1 mm was connected to a load sensor fixed on a linear motorized stage that was able to move the tip up and down with a high resolution of a few nm. The indentation depth was set to 3 µm. The loading and unloading processes were conducted at a speed of 0.1 µm/s. The reaction force with respect to indentation depth during loading and unloading was continuously detected by the load sensor in order to investigate the mechanical property of hydrogel coating. Using the data obtained from the indentation tests, hardness, elastic modulus and stiffness of the hydrogel coating were assessed^40–42^.

**Table 2:** Experimental conditions for indentation and friction/wear experiments.

Indentation experiment		Friction/wear experiment	
Loading force	Measurement value (mN)	Normal load	20, 30 mN
Indentation depth	~ 5 µm	Sliding stroke	1, 2, 4 mm
Loading speed	0.1 µm/s	Sliding cycle	90 cycles
Unloading speed	0.1 µm/s	Sliding speed	4 mm/s
Tip material	Stainless steel (SUS 304)	Tip material	ZrO_2_ ball
Ball tip diameter	1 mm	Ball tip diameter	1 mm

### Friction and wear experiments

A reciprocating-type sliding tester was used for the friction and wear tests. The sliding tester had two load sensors that operated simultaneously. One sensor measured the frictional force in the lateral direction and the other one measured the normal load in the vertical direction. Using a feedback control system, it was possible to maintain a precise normal load over the entire sliding cycle. For the counter tip, a zirconia ball with a diameter of 1 mm was attached to the suspension attached to the force sensor. The specific conditions of the friction and wear experiments are presented in Table 2. The normal load and the sliding stroke length were used as experimental variables. Sliding stroke lengths of 1, 2, and 4 mm were used. All the sliding tests were conducted in a Class 100 clean booth at room temperature and humidity. The sliding tests were repeated at least three times under the same conditions.

The frictional characteristics of the hydrogel coating specimen with respect to the normal load were observed and evaluated in real time during the sliding tests. The variation of frictional data with respect to the increase in the sliding stroke length during the same test was also monitored. The generation of the wear track was visualized by a 3D laser scanning confocal microscope installed above the tribotester. Moreover, the specific characteristics of the wear track formed on the surface of the hydrogel coating due to the repeated sliding motion was analyzed through SEM analysis. To induce the self-healing effect of the hydrogel coating, the wear track was hydrated with water, and the surface profiles of the wear tracks before and after hydration were compared. After naturally drying the hydrated hydrogel coating, the friction and wear characteristics were assessed with respect to the number of sliding cycles and the normal load.
